# *N*-(2-Chloro­benzo­yl)-*N*′-(3-pyrid­yl)thio­urea

**DOI:** 10.1107/S1600536808019922

**Published:** 2008-07-05

**Authors:** Lan-Qin Chai, Yu-Jie Ding, Xiao-Qing Yang, Hai-Bo Yan, Wen-Kui Dong

**Affiliations:** a School of Chemical and Biological Engineering, Lanzhou Jiaotong University, Lanzhou 730070, People’s Republic of China; b Department of Biochemical Engineering, Anhui University of Technology and Science, Wuhu 241000, People’s Republic of China

## Abstract

In the mol­ecule of the title compound, C_13_H_10_ClN_3_OS, the dihedral angles between the plane through the thio­urea group and the pyridine and benzene rings are 53.08 (3) and 87.12 (3)°, respectively. The mol­ecules are linked by inter­molecular N—H⋯N hydrogen-bonding inter­actions to form a supra­molecular chain structure along the *a* axis. An intra­mol­ecular N—H⋯O hydrogen bond is also present.

## Related literature

For related literature, see: Campo *et al.* (2002 ▶); Dong *et al.* (2006 ▶, 2008 ▶); Foss *et al.* (2004 ▶); Guillon *et al.* (1996 ▶); Koch (2001 ▶); Krepps *et al.* (2001 ▶); Su *et al.* (2004 ▶, 2006 ▶); Teoh *et al.* (1999 ▶); Venkatachalam *et al.* (2004 ▶); West *et al.* (2000 ▶); Xian *et al.* (2004 ▶).
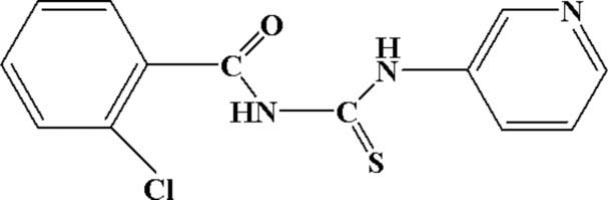


## Experimental

### 

#### Crystal data

C_13_H_10_ClN_3_OS
*M* *_r_* = 291.75
Triclinic, 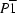

*a* = 8.421 (3) Å
*b* = 9.282 (4) Å
*c* = 10.512 (4) Å
α = 98.336 (4)°
β = 110.797 (4)°
γ = 112.532 (4)°
*V* = 670.9 (5) Å^3^
*Z* = 2
Mo *K*α radiation
μ = 0.43 mm^−1^
*T* = 298 (2) K
0.32 × 0.11 × 0.07 mm

#### Data collection

Bruker SMART 1000 CCD area-detector diffractometer
Absorption correction: multi-scan (*SADABS*; Sheldrick, 1996 ▶) *T*_min_ = 0.874, *T*_max_ = 0.972
3504 measured reflections
2319 independent reflections
1734 reflections with *I* > 2σ(*I*)
*R*_int_ = 0.019

#### Refinement

*R*[*F* ^2^ > 2σ(*F* ^2^)] = 0.036
*wR*(*F* ^2^) = 0.100
*S* = 1.02
2319 reflections
172 parameters
H-atom parameters constrained
Δρ_max_ = 0.20 e Å^−3^
Δρ_min_ = −0.18 e Å^−3^

### 

Data collection: *SMART* (Siemens, 1996 ▶); cell refinement: *SAINT* (Siemens, 1996 ▶); data reduction: *SAINT*; program(s) used to solve structure: *SHELXS97* (Sheldrick, 2008 ▶); program(s) used to refine structure: *SHELXL97* (Sheldrick, 2008 ▶); molecular graphics: *SHELXTL* (Sheldrick, 2008 ▶); software used to prepare material for publication: *SHELXTL*.

## Supplementary Material

Crystal structure: contains datablocks global, I. DOI: 10.1107/S1600536808019922/rz2227sup1.cif

Structure factors: contains datablocks I. DOI: 10.1107/S1600536808019922/rz2227Isup2.hkl

Additional supplementary materials:  crystallographic information; 3D view; checkCIF report

## Figures and Tables

**Table 1:** Hydrogen-bond geometry (Å, °)

*D*—H⋯*A*	*D*—H	H⋯*A*	*D*⋯*A*	*D*—H⋯*A*
N2—H2⋯O1	0.86	1.98	2.671 (3)	137
N1—H1⋯N3^i^	0.86	2.08	2.886 (4)	157

Symmetry code: (i) 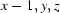.

**Fig. 1.**
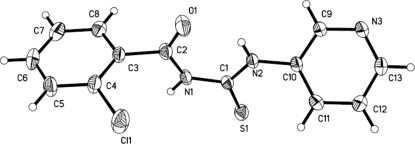
The molecule structure of the title compound with atom numbering. Displacement ellipsoids for non-hydrogen atoms are drawn at the 30% probability level.

**Fig. 2.**
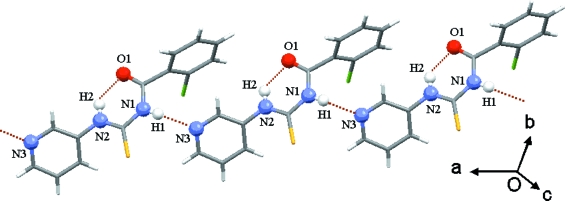
The supramolecular chain structure of the title compound constructed by intermolecular N—H···N hydrogen bonding interactions (dotted lines).

## supplementary crystallographic information

### Comment

Thiourea and its substituted derivatives have attracted much attention because of their unique properties, such as the strong coordination ability (Su *et al.*, 2004; Su *et al.*, 2006; Xian *et al.*, 2004; West *et al.*, 2000). They are used as selective analytical reagents, especially for the determination of transition metals in complex interfering matrices (Koch, 2001; Foss *et al.*, 2004). It has been shown that the redox properties of thiourea are markedly influenced by electronic factors (Guillon *et al.*, 1996), and the biological activity of thiourea derivatives has also been reported in the literature (Teoh *et al.*, 1999; Campo *et al.*, 2002). However, the study of S···H interactions may have fundamental importance in biochemical research due to the fact that living systems contain several important sulfur-containing molecules, such as the aminoacids cysteine and methionine (Krepps *et al.*, 2001). Related to the biological relevance of S···H interactions, Uckum and coworkers have recently reported a structural study of a series of thiourea compounds (Venkatachalam *et al.*, 2004). Here we report the synthesis and crystal structure of a new benzoylthiourea derivative, *N*-(*o*-chloro)benzoyl-*N*'-(3-pyridyl)thiourea. The molecular structure of the title compound is shown in Figure 1.

The dihedral angles formed by the plane through the thiourea group and the pyridine and benzene rings of 53.08 (3) and 87.12 (3)°, respectively. The molecular conformation is stabilized by an intramolecular N—H···O hydrogen bonding interaction (Table 1), forming a planar six-membered ring. In contrast to other thiourea compounds, the H1···S1 separation is 2.662 (2) Å, indicating that S1 is not involved in hydrogen bonding. This situation is similar to that found in the structure of *N*-benzoyl-*N*'-(3-pyridyl)thiourea (Dong *et al.*, 2006). The C=O bond length of 1.217 (3) Å is just significantly longer than the average C=O bond length (1.200 Å) due to the intramolecular hydrogen bond. In the crystal structure, molecules are linked by intermolecular by N—H···N hydrogen interactions to form supramolecular chains along the *a* axis.

### Experimental

*N*-(*o*-Chloro)benzoyl-*N*'-(3-pyridyl)thiourea was synthesized according the method reported in the literature (Dong *et al.*, 2008). *o*-Chlorobenzoyl chloride (3.61 g, 0.02 mol) was reacted with ammonium thiocyanate (2.28 g, 0.03 mol) in CH_2_Cl_2_ (25 ml) under solid-liquid phase transfer catalysis, using 3% polyethylene glycol-400 (0.36 g) as catalyst, to give the corresponding benzoyl isothiocyanate, which was reacted with 3-aminopyridine (1.72 g, 0.02 mol). The title compound precipitated immediately. The product was filtered, washed with water and CH_2_Cl_2_ and dried. Colourless needle-shaped single crystals were obtained by slow evaporation of an acetone solution after several weeks at room temperature. M.p. 442 - 444 K. Anal. Calcd. for C_13_H_10_ClN_3_OS: C, 53.52; H, 3.45; N, 14.40. Found: C, 53.28; H, 3.48; N, 14.15%.

### Refinement

H atoms were treated as riding atoms with C—H = 0.93 Å, N—H = 0.86 Å, and *U*_iso_(H) = 1.2 *U*_eq_(C, N).

### Crystal data

**Table:**

C_13_H_10_ClN_3_OS	*Z* = 2
*M**_r_* = 291.75	*F*_000_ = 300
Triclinic, *P*1	*D*_x_ = 1.444 Mg m^−^^3^
Hall symbol: -P 1	Mo *K*α radiation λ = 0.71073 Å
*a* = 8.421 (3) Å	Cell parameters from 1514 reflections
*b* = 9.282 (4) Å	θ = 2.5–26.6º
*c* = 10.512 (4) Å	µ = 0.43 mm^−^^1^
α = 98.336 (4)º	*T* = 298 (2) K
β = 110.797 (4)º	Needle, colourless
γ = 112.532 (4)º	0.32 × 0.11 × 0.07 mm
*V* = 670.9 (5) Å^3^	

### Data collection

**Table:**

Bruker SMART 1000 CCD area-detector diffractometer	2319 independent reflections
Radiation source: fine-focus sealed tube	1734 reflections with *I* > 2σ(*I*)
Monochromator: graphite	*R*_int_ = 0.019
*T* = 298(2) K	θ_max_ = 25.0º
φ and ω scans	θ_min_ = 2.2º
Absorption correction: multi-scan(SADABS; Sheldrick, 1996)	*h* = −10→9
*T*_min_ = 0.874, *T*_max_ = 0.972	*k* = −11→9
3504 measured reflections	*l* = −12→9

### Refinement

**Table:**

Refinement on *F*^2^	Secondary atom site location: difference Fourier map
Least-squares matrix: full	Hydrogen site location: inferred from neighbouring sites
*R*[*F*^2^ > 2σ(*F*^2^)] = 0.037	H-atom parameters constrained
*wR*(*F*^2^) = 0.100	*w* = 1/[σ^2^(*F*_o_^2^) + (0.044*P*)^2^ + 0.2146*P*] where *P* = (*F*_o_^2^ + 2*F*_c_^2^)/3
*S* = 1.02	(Δ/σ)_max_ < 0.001
2319 reflections	Δρ_max_ = 0.20 e Å^−^^3^
172 parameters	Δρ_min_ = −0.18 e Å^−^^3^
Primary atom site location: structure-invariant direct methods	Extinction correction: none

### Special details

**Table:**

Geometry. All e.s.d.'s (except the e.s.d. in the dihedral angle between two l.s. planes) are estimated using the full covariance matrix. The cell e.s.d.'s are taken into account individually in the estimation of e.s.d.'s in distances, angles and torsion angles; correlations between e.s.d.'s in cell parameters are only used when they are defined by crystal symmetry. An approximate (isotropic) treatment of cell e.s.d.'s is used for estimating e.s.d.'s involving l.s. planes.
Refinement. Refinement of *F*^2^ against ALL reflections. The weighted *R*-factor *wR* and goodness of fit *S* are based on *F*^2^, conventional *R*-factors *R* are based on *F*, with *F* set to zero for negative *F*^2^. The threshold expression of *F*^2^ > σ(*F*^2^) is used only for calculating *R*-factors(gt) *etc*. and is not relevant to the choice of reflections for refinement. *R*-factors based on *F*^2^ are statistically about twice as large as those based on *F*, and *R*- factors based on ALL data will be even larger.

### Fractional atomic coordinates and isotropic or equivalent isotropic displacement parameters (Å^2^)

**Table:**

	*x*	*y*	*z*	*U*_iso_*/*U*_eq_	
Cl1	0.41414 (14)	0.64074 (11)	0.50918 (8)	0.0781 (3)	
N1	0.4306 (3)	0.5361 (2)	0.1841 (2)	0.0397 (5)	
H1	0.3077	0.4966	0.1477	0.048*	
N2	0.6871 (3)	0.4903 (2)	0.2029 (2)	0.0416 (5)	
H2	0.7561	0.5927	0.2548	0.050*	
N3	1.0366 (3)	0.3871 (2)	0.1394 (2)	0.0481 (5)	
O1	0.7112 (3)	0.7734 (2)	0.3311 (2)	0.0630 (5)	
S1	0.33789 (9)	0.24252 (8)	0.02568 (7)	0.0500 (2)	
C1	0.4967 (3)	0.4294 (3)	0.1432 (2)	0.0370 (5)	
C2	0.5364 (4)	0.6956 (3)	0.2748 (3)	0.0422 (6)	
C3	0.4139 (3)	0.7708 (3)	0.2962 (2)	0.0400 (5)	
C4	0.3540 (4)	0.7557 (3)	0.4027 (3)	0.0476 (6)	
C5	0.2449 (4)	0.8281 (3)	0.4233 (3)	0.0560 (7)	
H5	0.2062	0.8177	0.4955	0.067*	
C6	0.1944 (4)	0.9154 (3)	0.3359 (3)	0.0613 (8)	
H6	0.1211	0.9644	0.3490	0.074*	
C7	0.2513 (4)	0.9307 (3)	0.2296 (3)	0.0593 (7)	
H7	0.2157	0.9895	0.1705	0.071*	
C8	0.3611 (4)	0.8595 (3)	0.2093 (3)	0.0495 (6)	
H8	0.3997	0.8711	0.1371	0.059*	
C9	0.9299 (3)	0.4610 (3)	0.1477 (2)	0.0411 (6)	
H9	0.9551	0.5586	0.1259	0.049*	
C10	0.7848 (3)	0.3985 (3)	0.1872 (2)	0.0380 (5)	
C11	0.7442 (4)	0.2524 (3)	0.2178 (3)	0.0481 (6)	
H11	0.6456	0.2070	0.2436	0.058*	
C12	0.8523 (4)	0.1757 (3)	0.2093 (3)	0.0542 (7)	
H12	0.8291	0.0776	0.2300	0.065*	
C13	0.9953 (4)	0.2463 (3)	0.1697 (3)	0.0537 (7)	
H13	1.0674	0.1930	0.1636	0.064*	

### Atomic displacement parameters (Å^2^)

**Table:**

	*U*^11^	*U*^22^	*U*^33^	*U*^12^	*U*^13^	*U*^23^
Cl1	0.1271 (8)	0.0975 (6)	0.0704 (5)	0.0861 (6)	0.0617 (5)	0.0496 (5)
N1	0.0341 (10)	0.0395 (11)	0.0488 (11)	0.0192 (9)	0.0225 (9)	0.0076 (9)
N2	0.0372 (11)	0.0368 (10)	0.0569 (12)	0.0195 (9)	0.0271 (10)	0.0101 (9)
N3	0.0439 (12)	0.0496 (12)	0.0650 (14)	0.0268 (10)	0.0339 (11)	0.0184 (11)
O1	0.0406 (11)	0.0539 (11)	0.0758 (13)	0.0192 (9)	0.0209 (10)	−0.0069 (10)
S1	0.0487 (4)	0.0416 (4)	0.0571 (4)	0.0206 (3)	0.0259 (3)	0.0051 (3)
C1	0.0437 (14)	0.0408 (13)	0.0405 (13)	0.0244 (11)	0.0273 (11)	0.0160 (10)
C2	0.0426 (15)	0.0419 (13)	0.0447 (14)	0.0219 (12)	0.0227 (12)	0.0074 (11)
C3	0.0408 (13)	0.0356 (12)	0.0459 (14)	0.0206 (11)	0.0213 (11)	0.0071 (11)
C4	0.0624 (17)	0.0491 (14)	0.0487 (14)	0.0376 (13)	0.0297 (13)	0.0177 (12)
C5	0.0712 (19)	0.0653 (17)	0.0544 (16)	0.0448 (16)	0.0386 (15)	0.0170 (14)
C6	0.0686 (19)	0.0596 (17)	0.0696 (19)	0.0464 (16)	0.0307 (16)	0.0129 (15)
C7	0.0712 (19)	0.0516 (16)	0.0672 (18)	0.0404 (15)	0.0291 (16)	0.0236 (14)
C8	0.0577 (16)	0.0438 (14)	0.0540 (15)	0.0264 (13)	0.0293 (13)	0.0168 (12)
C9	0.0374 (13)	0.0396 (13)	0.0515 (14)	0.0198 (11)	0.0238 (12)	0.0142 (11)
C10	0.0388 (13)	0.0410 (13)	0.0432 (13)	0.0231 (11)	0.0236 (11)	0.0120 (10)
C11	0.0507 (15)	0.0512 (15)	0.0599 (16)	0.0269 (13)	0.0380 (13)	0.0222 (13)
C12	0.0630 (17)	0.0487 (15)	0.0750 (18)	0.0351 (14)	0.0422 (15)	0.0300 (14)
C13	0.0556 (16)	0.0537 (16)	0.0732 (18)	0.0373 (14)	0.0378 (15)	0.0220 (14)

### Geometric parameters (Å, °)

**Table:**

Cl1—C4	1.734 (2)	C5—C6	1.372 (4)
N1—C2	1.368 (3)	C5—H5	0.9300
N1—C1	1.394 (3)	C6—C7	1.369 (4)
N1—H1	0.8600	C6—H6	0.9300
N2—C1	1.331 (3)	C7—C8	1.380 (4)
N2—C10	1.422 (3)	C7—H7	0.9300
N2—H2	0.8600	C8—H8	0.9300
N3—C13	1.335 (3)	C9—C10	1.375 (3)
N3—C9	1.340 (3)	C9—H9	0.9300
O1—C2	1.217 (3)	C10—C11	1.381 (3)
S1—C1	1.659 (2)	C11—C12	1.371 (3)
C2—C3	1.508 (3)	C11—H11	0.9300
C3—C4	1.385 (3)	C12—C13	1.373 (3)
C3—C8	1.386 (3)	C12—H12	0.9300
C4—C5	1.384 (3)	C13—H13	0.9300
			
C2—N1—C1	128.3 (2)	C7—C6—H6	119.8
C2—N1—H1	115.9	C5—C6—H6	119.8
C1—N1—H1	115.9	C6—C7—C8	120.5 (2)
C1—N2—C10	124.83 (19)	C6—C7—H7	119.7
C1—N2—H2	117.6	C8—C7—H7	119.7
C10—N2—H2	117.6	C7—C8—C3	120.1 (2)
C13—N3—C9	117.2 (2)	C7—C8—H8	119.9
N2—C1—N1	115.4 (2)	C3—C8—H8	119.9
N2—C1—S1	125.54 (17)	N3—C9—C10	122.6 (2)
N1—C1—S1	119.02 (17)	N3—C9—H9	118.7
O1—C2—N1	124.8 (2)	C10—C9—H9	118.7
O1—C2—C3	122.1 (2)	C9—C10—C11	119.2 (2)
N1—C2—C3	113.1 (2)	C9—C10—N2	119.10 (19)
C4—C3—C8	118.6 (2)	C11—C10—N2	121.6 (2)
C4—C3—C2	121.6 (2)	C12—C11—C10	118.6 (2)
C8—C3—C2	119.8 (2)	C12—C11—H11	120.7
C5—C4—C3	121.1 (2)	C10—C11—H11	120.7
C5—C4—Cl1	119.9 (2)	C11—C12—C13	118.8 (2)
C3—C4—Cl1	118.97 (18)	C11—C12—H12	120.6
C6—C5—C4	119.2 (2)	C13—C12—H12	120.6
C6—C5—H5	120.4	N3—C13—C12	123.6 (2)
C4—C5—H5	120.4	N3—C13—H13	118.2
C7—C6—C5	120.4 (2)	C12—C13—H13	118.2
			
C10—N2—C1—N1	173.77 (19)	C4—C5—C6—C7	0.0 (4)
C10—N2—C1—S1	−7.1 (3)	C5—C6—C7—C8	−0.4 (4)
C2—N1—C1—N2	1.6 (3)	C6—C7—C8—C3	0.4 (4)
C2—N1—C1—S1	−177.58 (18)	C4—C3—C8—C7	0.0 (4)
C1—N1—C2—O1	3.2 (4)	C2—C3—C8—C7	−179.1 (2)
C1—N1—C2—C3	−178.6 (2)	C13—N3—C9—C10	0.8 (4)
O1—C2—C3—C4	−93.6 (3)	N3—C9—C10—C11	−1.0 (4)
N1—C2—C3—C4	88.1 (3)	N3—C9—C10—N2	175.7 (2)
O1—C2—C3—C8	85.4 (3)	C1—N2—C10—C9	129.9 (2)
N1—C2—C3—C8	−92.8 (3)	C1—N2—C10—C11	−53.4 (3)
C8—C3—C4—C5	−0.4 (4)	C9—C10—C11—C12	0.8 (4)
C2—C3—C4—C5	178.7 (2)	N2—C10—C11—C12	−175.8 (2)
C8—C3—C4—Cl1	178.40 (18)	C10—C11—C12—C13	−0.5 (4)
C2—C3—C4—Cl1	−2.5 (3)	C9—N3—C13—C12	−0.5 (4)
C3—C4—C5—C6	0.4 (4)	C11—C12—C13—N3	0.4 (4)
Cl1—C4—C5—C6	−178.4 (2)		

### Hydrogen-bond geometry (Å, °)

**Table:**

*D*—H···*A*	*D*—H	H···*A*	*D*···*A*	*D*—H···*A*
N2—H2···O1	0.86	1.98	2.671 (3)	137
N1—H1···N3^i^	0.86	2.08	2.886 (4)	157

Symmetry codes: (i) *x*−1, *y*, *z*.
